# Application of image processing and transfer learning for the detection of rust disease

**DOI:** 10.1038/s41598-023-31942-9

**Published:** 2023-03-29

**Authors:** Fereshteh Shahoveisi, Hamed Taheri Gorji, Seyedmojtaba Shahabi, Seyedali Hosseinirad, Samuel Markell, Fartash Vasefi

**Affiliations:** 1 Department of Plant Pathology, North Dakota State University, Fargo, ND USA; 2 Department of Plant Sciences and Landscape Architecture, University of Maryland, College Park, MD USA; 3 Biomedical Engineering Program, College of Engineering and Mine, University of North Dakota, Grand Forks, ND USA; 4 School of Electrical Engineering and Computer Science, College of Engineering and Mine, University of North Dakota, Grand Forks, ND USA; 5 Department of Plant Sciences, North Dakota State University, Fargo, ND USA; 6 SafetySpect Inc., 10100 Santa Monica Blvd., Suite 300, Los Angeles, CA USA

**Keywords:** Biotic, Plant physiology

## Abstract

Plant diseases introduce significant yield and quality losses to the food production industry, worldwide. Early identification of an epidemic could lead to more effective management of the disease and potentially reduce yield loss and limit excessive input costs. Image processing and deep learning techniques have shown promising results in distinguishing healthy and infected plants at early stages. In this paper, the potential of four convolutional neural network models, including Xception, Residual Networks (ResNet)50, EfficientNetB4, and MobileNet, in the detection of rust disease on three commercially important field crops was evaluated. A dataset of 857 positive and 907 negative samples captured in the field and greenhouse environments were used. Training and testing of the algorithms were conducted using 70% and 30% of the data, respectively where the performance of different optimizers and learning rates were tested. Results indicated that EfficientNetB4 model was the most accurate model (average accuracy = 94.29%) in the disease detection followed by ResNet50 (average accuracy = 93.52%). Adaptive moment estimation (Adam) optimizer and learning rate of 0.001 outperformed all other corresponding hyperparameters. The findings from this study provide insights into the development of tools and gadgets useful in the automated detection of rust disease required for precision spraying.

## Introduction

‘Rust’ fungi (order Pucciniales, division Basidiomycota) are a major group of fungal plant pathogens that can affect the yield and quality of many field crops, including sunflower, soybean, field pea, dry bean, wheat, and barley. The level of yield loss caused by rust fungi varies among the host species. For instance, wheat leaf rust (*Puccinia triticina* Eriks.) can cause yield losses ranging between 3 to 50% depending on the geographical location^1^. Soybean rust (*Phakopsora pachyrhizi* Syd. & P. Syd) usually cause sporadic yield losses in the United States; however, according to risk analyses, the pathogen can result in yield losses greater than 10% in any soybean growing region in the US while the losses can reach up to 50% in southeastern states^2^. Sunflower rust (*Puccinia* *helianthi* Schwein) is one of the important diseases that limit sunflower yield. A comprehensive study conducted by Friskop et al. indicated that every 1% of disease severity could result in 6.6% yield reduction; however, yield losses up to 80% have been reported under a high disease severity^3^.

Three important rust diseases of field crops grown in the US Northern Great Plains include; sunflower rust, common bean rust (*Uromyces appendiculatus* F. Strauss), and field pea rust (*Uromyces viciae-fabae* (Pers.) de Bary) that affect sunflower (*Helianthus annuus* L.), dry bean (*Phaseolus vulgaris* L.), and field pea (*Pisum* *sativum* L.), respectively. These three rust pathogens have five spore stages (macrocyclic rust); while some of the spore stages are morphologically similar among the hosts, there are differences in signs of each pathogen and symptoms of the disease on hosts. In the first stage, basidiospores infect the plant and produce pycnia; these spores are not visible with the unaided eye; the earliest visible stage is the pycnial stage. On sunflower, pycnia appear as small yellow-orange spots on the top side of lower leaves and cotyledons. In the next stage, aecia will form in clusters of orange cups (approximately 0.5 cm in size) on the underside of the leaf. The most common and repeating stage of rust is uredinia which occurs after aecia. Uredinia are small pustules (approximately 0.15 cm) filled with cinnamon-brown spores (urediniospores) and appear on the upper or undersides of leaves. On sunflower, Urediniospores can infect stems, bracts, leaves, and petioles. These pustules can be rubbed off easily and may be surrounded by a chlorotic halo. Lastly, uredinia turn black and form black structures (telia) that could survive under unfavorable environmental conditions^4^. On dry bean, yellow to yellow–brown pycnia form on the upper surface of leaves and white aecia appear on the underside of leaves. Similar to sunflower rust, pycnia and aecia are difficult to detect and last only a few days. The visible symptoms start with small white or yellow raised spots on the upper or/and undersides of leaves. These spots enlarge and form reddish-brown uredinia that are about < 0.1–0.3 cm in diameter that are filled with dusty cinnamon-brown spores. Pustules may form on green pods, and occasionally on branches and stems and they may be surrounded by chlorotic halos. Premature leaf drop may be observed under high disease pressure^5^. On field pea, small whitish aecia (0.3–0.4 mm diameter) may be scattered on tissue or in groups surrounding the pycnia. Aecia enlarge and rupture the epidermis to produce uredinia (0.5–1 + mm diameter) filled with cinnamon-brown which could form on leaves, petioles, stems, and pods^6^. Details about the morphology of each rust pathogen and its specific host play a significant role in the early detection and management of the epidemic, which could prevent yield losses on several host species.

An integrated pest management (IPM) approach is commonly recommended for managing rust diseases. This includes planting a cultivar with genetics conferring resistance to the rust pathogen(s), timely application of an efficacious fungicide, crop rotation, excellent control of volunteer and or wild host species^7,8^. Accurate identification and on-time detection of the disease could increase the efficiency of the management practices. Growers need to have ample tools and knowledge to identify a disease, which is not always possible. Further, detection of the all the infested areas in the field is not practical; therefore, growers consider spraying the whole field, regardless of the distribution and spread of the disease in the field “(personal communication with growers)”. Considering this challenge, tools that make disease identification and detection easier would be valuable for plant disease management. Automatic detection of diseases through machine learning could provide timely and accurate detection of plant diseases and could be used to spot the infected areas in the field and apply pesticides only on those areas^9^. Precise spraying could significantly minimize unnecessary pesticide applications.

Numerous studies have verified the efficiency of machine learning analyses in plant pathology. These studies are either focused on training disease prediction models using environmental factors (i.e., temperature, humidity, and wetness duration)^9–12^ or the detection of plant diseases using image processing and machine learning^13^. The high accuracy of conventional machine learning algorithms such as artificial neural network (ANN), random forest (RF), support vector regression (SVR), multi-layer perceptron (MLP), extreme learning machine (ELM), and logistic regression (LR) in distinguishing healthy and infected samples has been reported repeatedly^9^. For example, Zhu et al.^14^ assessed the accuracy of different machine learning algorithms, including back-propagation neural network (BPNN), ELM, and least squares support vector machine (LS-SVM) in the detection of *Tobacco mosaic virus* using hyperspectral imaging. The majority of these models showed prediction accuracies over 85%.

In an earlier study conducted by Rumpf et al.^15^ the accuracy of ANN, support vector machine (SVM), and decision trees (DT) in the classification of healthy and inoculated sugar beet leaves with three diseases (Cercospora leaf spot, rust, and powdery mildew) was evaluated. Results of this work revealed that SVM accuracy in the detection of Cercospora leaf spot disease increased from 65% at 1–2% disease severity to 100% at 10% disease severity. The range of accuracy was similarly high for the other two tested diseases. While the classification accuracy of the conventional machine learning methods is promising, they require extracting meaningful information (feature extraction) from the input data. Feature extraction is an extra computational step, and the performance of the machine learning models depends on the type of extracted information. Convolutional neural network (CNN) models developed in recent years are capable of processing raw data directly and extracting the efficient features automatically^16,17^. Further, CNNs could result in higher classification accuracies in comparison with traditional machine learning algorithms^18^.

Efficiency of CNN models has been reported in different fields such as medical sciences^19–21^, food industry^22^, construction industry^23^, weather prediction^24^, advertisement^25^, and hydrology^26^. Application of CNN models in the detection of plant diseases has been studied to some extent. A review paper published by Boulent et al.^27^ reported the results of several studies where the accuracy of CNN and traditional image processing methods in the prediction of plant diseases were compared. As a general trend, CNN outperformed models such as SVM and radial basis function (RBF) with differences in accuracy ranging between 3 and 29%^28,29^. Another significant strength of CNNs is their high generalization capacity (how accurate a model can classify or predict previously unseen data) which results in increased robustness even when the data is heterogeneous, the image capturing conditions are different, and there are variabilities among classes. However, acquiring this robustness requires a large-scale training dataset^27^ which is not always available when researchers tend to use their own dataset. Transfer learning models have become a reliable alternative to CNNs regardless of the training dataset size.

Pre-trained models such as EfficientNet and MobileNet, with varying depth layers, have been used in the classification of plant diseases^30–37^. Wang et al.^36^ compared the accuracy of shallow networks and three deep models in the classification of apple black rot images extracted from the PlantVillage dataset. Results of this study indicated that VGG16 (VGGNet with 16 weight layers) from the deep models outperformed other tested models, where the overall accuracy was 90.4% on the test dataset. In another study conducted by Zhang et al.^37^, the accuracy of three models, including AlexNet, GoogLeNet, and ResNet, with different optimization methods (i.e., Stochastic Gradient Descent (SGD) and Adam) were evaluated. Most of the tested models showed accuracies greater than 94%, with ResNet_SGD resulting in the highest accuracy of 97.28%. These studies and several other reports have verified the efficiency of transfer learning in the detection of plant diseases. However, only limited studies have been conducted on the rust disease(s) using images from larger datasets such as PlantVillage. These images are commonly captured under controlled environments including homogeneous backgrounds, fixed light intensity and tissue/camera positions^38,39^. In other studies models were developed to detect rust using only one host crop^40,41^; this could limit the model generalization when is used for the disease detection in other crops. The present study was designed to fill the aforementioned gaps and indicate the application of transfer learning in the detection of rust disease using real-life images taken under field conditions. Supplementary Table S1 provides comparisons between the methodologies and results of our work and those of previously published studies reporting top classifiers and CNN models in the classification of binary (healthy vs infected) and multiple-class (several diseases) datasets. The objectives were (i) to evaluate the accuracy of four deep CNN models, including Xception, ResNet50, EfficientNet, and MobileNet, in the classification of images taken under field and greenhouse conditions into healthy and infected leaves (that displayed uredinia pustules of *Puccinia*. sp or *Uromyces.* Spp.; hereafter, “detection of rust disease” will refer to “detection of uredinia pustules”) of three economically important field crops (i.e., sunflower, dry bean, and field pea). (ii) to assess the role of different optimizers and learning rates in the performance of the models.

## Results and discussion

The results of the transfer learning analyses conducted with different hyperparameters using images captured in greenhouse and field conditions are presented and discussed in this section.

The performance of four pre-trained models including ResNet, Xception, EfficientNetB4, and MobileNetV2 in the detection of rust disease on three hosts was evaluated using four commonly used optimizers and three different learning rates. In general, EfficientNetB4 with an average accuracy of 94.29% across all learning rates and optimizers was the most efficient model in distinguishing healthy and infected leaf tissues in all three hosts. The average accuracy of the ResNet50 model across all hyperparameters was only 0.77% less than EfficientNetB4 and was the second-best model (average accuracy = 93.52%). The average accuracies of MobileNet-V2 and Xception were 87.67 and 83.20% using different hyperparameters (Tables 1 and 2). The higher accuracy of the EfficientNetB4 model could be due to its architecture that not only balances the network dimension in terms of depth, width, and resolution of the input image but uses squeeze-and-excitation that enhance the representational power of the network^42^. The application and high efficiency of the EfficientNet model in the detection of other plant diseases have been reported previously^31,38,43^.

**Table 1:** Statistical fitness metrics of the residual network (ResNet), Xception, EfficientNetB4, and MobileNetV2 pre-trained conventional neural network (CNN) models in the detection of rust disease on sunflower, dry bean, and field pea using Adaptive Moment Estimation (Adam), Follow The Regularized Leader (Ftrl), Stochastic Gradient Descent (SGD), and Root Mean Square Propagation (RMSprop) optimizers.

Model	Optimizer	Accuracy	Precision	True positive rate	True negative rate	F-score	AUC-ROC
ResNet50	Adam	94.54	96.25	92.70	96.39	94.44	96.00
	SGD	93.94	95.44	91.73	95.97	93.54	94.52
	RMSprop	92.81	94.82	89.51	95.67	92.04	93.70
	Ftrl	92.22	94.45	89.75	94.68	92.01	92.97
EfficientNetB4	Adam	95.56	96.86	93.65	97.25	95.18	96.92
	SGD	94.49	95.45	92.53	96.19	93.96	95.56
	RMSprop	92.95	94.04	90.63	94.95	92.25	94.20
	Ftrl	92.96	95.26	89.74	95.91	92.39	93.67
Xception	Adam	84.84	86.97	82.34	87.35	84.45	86.00
	SGD	84.07	82.79	83.64	84.44	82.98	84.30
	RMSprop	82.44	82.16	80.62	84.02	80.81	83.91
	Ftrl	83.54	83.13	82.41	84.58	82.69	84.32
MobileNet-V2	Adam	88.71	90.09	87.04	90.39	88.52	89.92
	SGD	87.59	89.75	82.99	91.56	86.09	88.66
	RMSprop	86.35	88.41	82.47	89.71	84.72	86.71
	Ftrl	87.29	88.95	83.52	90.55	85.90	88.19

AUC-ROC = area under the receiver operating characteristic curve. All units are in percentage.

**Table 2:** Statistical fitness metrics of the residual network (ResNet), Xception, EfficientNetB4, and MobileNetV2 pre-trained conventional neural network (CNN) models in the detection of rust disease on sunflower, dry bean, and field pea using 0.01, 0.001, and 0.0001 learning rates.

Model	Learning rate	Accuracy	Precision	True positive rate	True negative rate	F-score	AUC-ROC
ResNet50	0.01	93.38	95.40	91.25	95.51	93.24	94.76
	0.001	94.54	96.25	92.70	96.39	94.44	96.00
	0.0001	93.20	94.37	91.67	94.67	92.95	93.86
EfficientNetB4	0.01	94.32	95.46	92.65	95.84	93.97	94.37
	0.001	95.56	96.86	93.65	97.25	95.18	95.92
	0.0001	94.21	95.14	92.29	95.88	93.66	95.62
Xception	0.01	80.85	84.48	76.53	85.18	79.98	82.25
	0.001	84.84	86.97	82.34	87.35	84.45	86.00
	0.0001	81.81	86.30	75.79	87.83	80.63	82.22
MobileNet-V2	0.01	86.36	88.31	84.89	87.83	86.03	87.31
	0.001	88.71	90.09	87.04	90.39	88.52	89.92
	0.0001	88.65	86.39	90.01	87.45	88.15	89.31

AUC-ROC = area under the receiver operating characteristic curve. All units are in percentage.

In a study conducted by Atila et al.^38^, the performance of several EfficientNet models, ResNet50, AlexNet, VCG16, and InceptionV3 using the PlantVillage dataset was assessed. The learning rate was set to 0.001 and 0.01 for Adam and SGD optimizers, respectively. The results indicated that the EfficientNetB4 model was the most accurate model in the detection of disease using the augmented data with an accuracy of 99.97% and EfficientNetB5 outperformed all other models (average accuracy = 99.91%) where the original dataset was used. However, the accuracy of other models in their study was very close to the top models; ranging from 99.45% for AlexNet in the original data set to 99.88% for ResNet50 in the augmented dataset. The previous studies that have reported the high efficiency of the EfficientNet model in the detection of plant diseases have mostly used PlantVillage which is a large publicly available dataset with edited images that have similar backgrounds and magnitudes. The result of the present study indicated that EfficientNet could be a good choice even for small datasets where a variety of images with different backgrounds, ambient light intensities, angles, and ages (for sunflower). Identifying models that perform well with these types of images is essential since scientists normally encounter such datasets in a real-life situation where acquired photos have complex background noise that makes the data analysis more challenging^18^.

Perusing literature indicated that ResNet50 models are among the most accurate and frequently used models in the detection of plant diseases. Several studies have reported the high efficiency of ResNet models where EfficientNet was not among the tested models^34,37,44–46^. Our results indicated that ResNet50 was the second best model in the detection of rust disease, with slightly lower accuracy than EfficientNet. Therefore, it could be concluded that EfficientNet and ResNet are two of the strongest pre-trained CNN models in the detection of plant diseases and pests. However, as previously reported^37,38,43^, model hyperparameters such as optimizers, learning rates, and batch size are highly determinant components in the performance of the models where the appropriate selection of these components in the training of a model warrants the highest accuracy.

**Figure 1:**
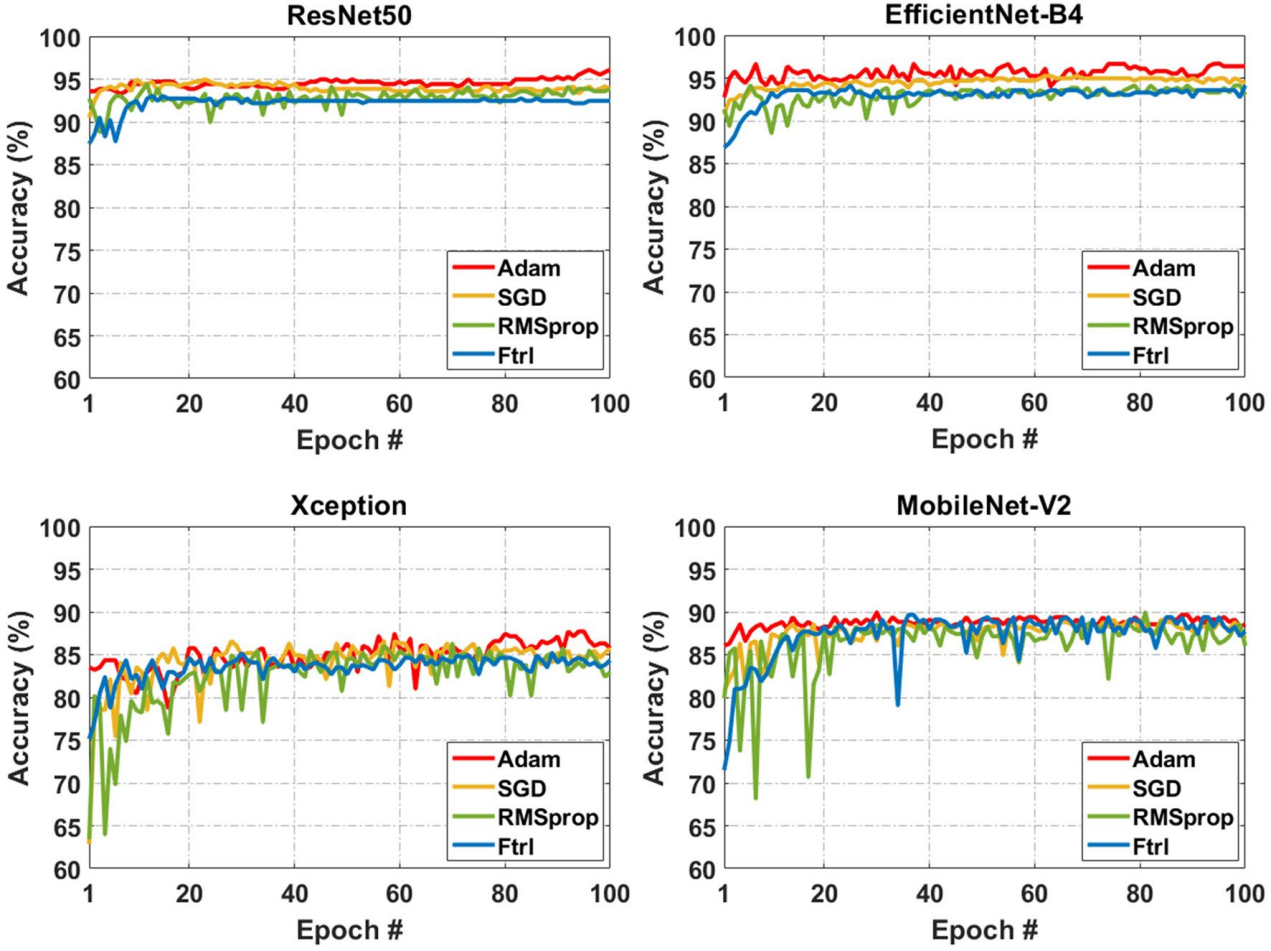
The effect of Adaptive Moment Estimation (Adam), Follow The Regularized Leader (Ftrl), Stochastic Gradient Descent (SGD), and Root Mean Square Propagation (RMSprop) optimizers with the learning rate of 0.001 on the accuracy of residual networks (ResNet), Xception, EfficientNetB4, and MobileNetV2 pre-trained conventional neural network (CNN) models using different epoch numbers.

Considering the significant role of the optimizer in training the deep learning models^47^, the effect of four optimizers on the performance of models was evaluated. Fitness metrics of ResNet, Xception, EfficientNetB4, and MobileNetV2 models were tested using the four most widely used optimizers including Adam^48^, Follow The Regularized Leader (Ftrl)^49^, SGD, and Root Mean Square Propagation (RMSprop)^50^. The results indicated that in general, the accuracy of models was higher with Adam optimizer, ranging between 84.84 and 95.56%. EfficientNetB4 with Adam optimizer and learning rate of 0.001 had an accuracy of 95.56%, precision of 96.86%, true positive rate of 93.65%, F-score of 95.18%, true negative rate of 97.25%, and the area under the receiver operating characteristic curve (AUC-ROC) of 96.92% and outperformed other models. Stochastic gradient descent was the second-best optimizer (after Adam) which resulted in an accuracy of 94.49% in EfficientNetB4. Root Mean Square Propagation and Ftrl were the least efficient optimizers and generated similar results in ResNet, Xception, and EfficientNetB4; however, the accuracy of MobileNetV2 decreased only by 0.3% when SGD was replaced with Ftrl. The details of the models’ fitness metrics are presented in Table 1. While the EfficientNetB4 model using the Adam optimizer outperformed the ResNet50 and the other models, the ResNet model was more stable with less fluctuation than the EfficientNetB4 regardless of epoch numbers (Fig. 1).

In a study conducted by Zhang et al.^37^, SGD optimizer showed a better performance than Adam where AlexNet, GoogLeNet, and ResNet models were used. While the difference in accuracy between the optimizers was only 1.60 and 2.12% for GoogLeNet and ResNet, respectively, the accuracy declined by 81.97% when SGD was substituted with Adam in AlexNet. In the present study, Adam was a better optimizer for all models. However, only one model was common (ResNet) between the studies where the difference in accuracy was less than 2.6% when the two optimizers were used in either of the studies. This suggests that the efficiency of optimizers could be model and hyperparameter-dependent to some degree. Further, the epoch number was different in these studies; Zhang et al.^37^ trained their model using 6240 epochs while we set the epoch to 100, and usually SGD could converge better than Adam with a longer training time^51^.

**Figure 2:**
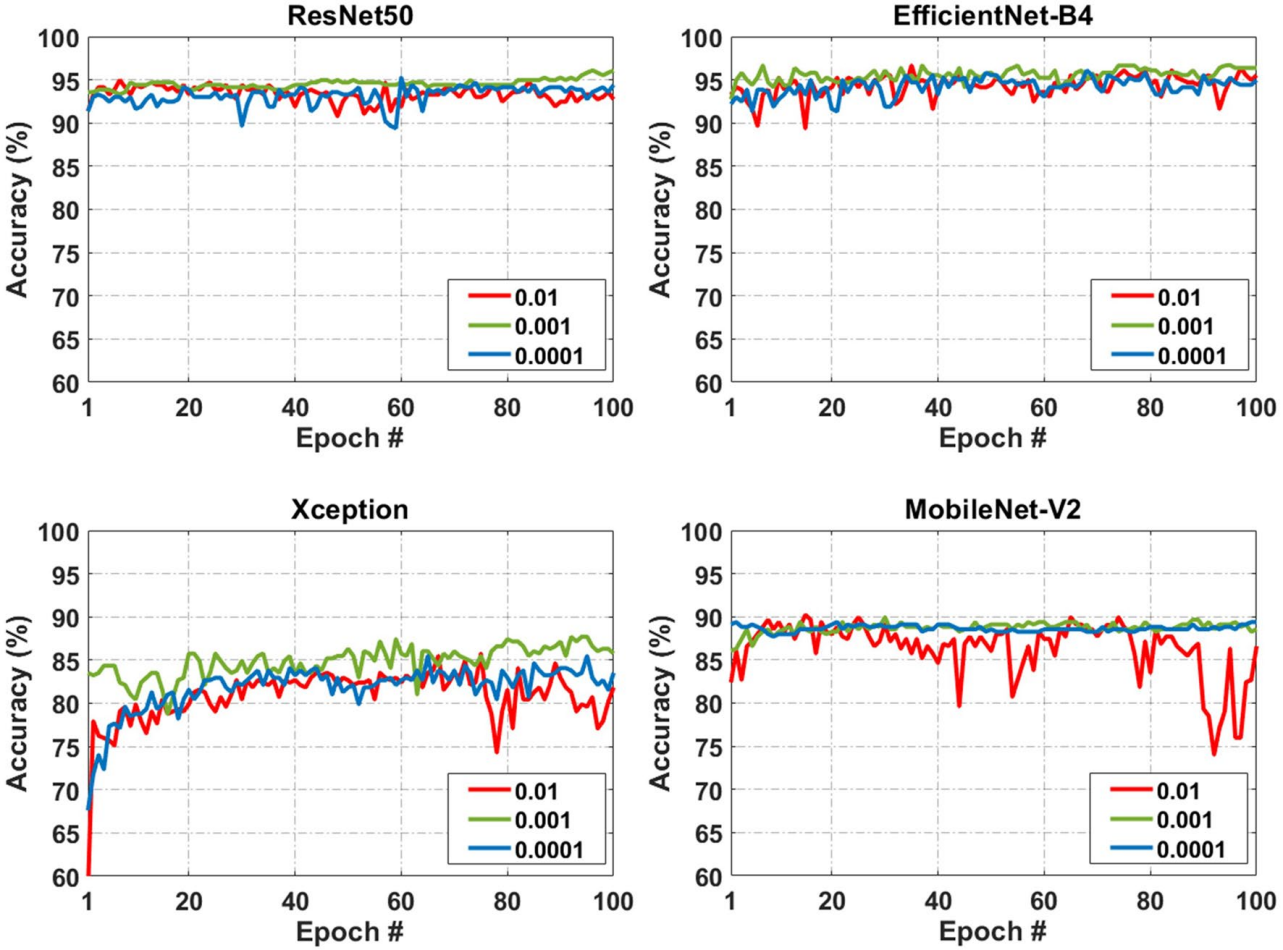
The effect of different learning rates (0.01, 0.001, and 0.0001) on the accuracy of the residual networks (ResNet), Xception, EfficientNetB4, and MobileNetV2 pre-trained conventional neural network (CNN) models using different epoch numbers where Adaptive Moment Estimation (Adam) was used as the optimizer of the models.

Learning rate is considered one of the most important hyperparameters that significantly impact the performance of CNN models^52^. Defining the optimum learning rate results in the highest performance of CNN models. Defining very small learning rates applies smaller changes to the weights and minimizes the model loss function; however, the model needs more epochs to learn the task. Selecting a high learning rate, on the other hand, speeds up the training process but it can increase the potential of generating unwanted divergent behavior in the model loss function^53^. Therefore, in this study, three mid-range learning rates (0.01, 0.001, and 0.0001) were used in the training and testing of the models and their effects on the performance of the models were evaluated. Considering that Adam was the best optimizer for all models, it was used in analyses conducted to test the learning rates. Results of this section indicated that the learning rate of 0.001 was optimum for all the four pre-trained CNN models regardless of the epoch number (Table 2 and Fig. 2). EfficientNetB4 represented the highest accuracy (average accuracy = 94.70%) across all learning rates where the learning rate of 0.001 resulted in the maximum accuracy of 95.56%, precision of 96.86%, true positive rate of 93.65%, and true negative rate of 97.25%. ResNet50 was the second-best model regardless of the learning rate (average accuracy = 93.71%). The average accuracy of Xception and MobileNet-V2 were 82.5% and 87.91% across all learning rates (Table 2).

A closer look at Fig. 2 reveals that the optimum learning rate of 0.001 resulted in the highest accuracy compared to the other two learning rates and the fluctuation in percentage of accuracy minimizes after 20 epochs at this learning rate. Increasing or decreasing the learning rate led to the weaker performance of the models. The smallest value for the learning rate (0.0001 in this study) resulted in a slightly better performance than the highest value (0.01) for Xception and MobileNet-V2 (epochs of 100); however, the learning rate of 0.01 resulted in negligibly higher accuracy compared to 0.0001 in ResNet50 and EfficientNetB4 models. In general, EfficientNetB4 and ResNet50 were more robust to the change of the learning rate values, whereas Xception and MobileNet were more sensitive to either decreasing or increasing the learning rate.

The role of different learning rates on the performance of CNN models has been studied to some extent. For instance, Hassan et al.^43^ tested a range of learning rates (0.01 to 0.0001) for their efficiency in training of several models including EfficientNetB0, MobileNetV2, InceptionV3, and InceptionResNetV2 using PlantVillage dataset; however, results of this study did not include the optimum learning rate used for each model. In another study conducted on PlantVillage and Nepal datasets, testing three learning rates (0.001, 0.0001, 0.00001) resulted in accuracies ranging between 99 to 100% in the CNN model and 81 to 100% in the Capsule Neural Network where the optimum learning rate of 0.0001 was reported as a result^54^. Our results did not agree with this finding where our optimum rate was 0.001. However, several factors such as the model architecture, size and type of dataset, number of disease classes, and other hyperparameters are determinants in the performance of the models and these factors were not all similar between the two studies and might be the potential reasons of this discrepancy. Therefore, evaluating a range of hyperparameters, such as learning rate and optimizer, is an important task to tune the model parameters and obtain the maximum accuracy for the desired dataset.

Convolutional neural network models are becoming popular tools in the detection of plant diseases. The result of the present study indicated that EfficientNetB4 with the average accuracy of 94.29% outperformed the other models. ResNet50 (average accuracy of 93.52%) was the second-best architecture in the detection of rust disease on sunflower, dry bean, and field pea where a small dataset with uneven backgrounds and magnitudes were used. Further, four different optimizers and three learning rates were tested across all architectures; Adam optimizer and learning rate of 0.001 consistently performed better in all evaluated architectures. Moreover, k-fold cross validation (tenfold) on EfficientNetB4 with the Adam optimizer and learning rate of 0.001 was used to assess the effect of bias caused by random train-test split. The model achieved an average accuracy of 94.34% ± 0.010, precision of 96.09% ± 0.006, true positive rate of 93.37% ± 0.018, true negative rate of 97.22% ± 0.005, and F-score of 94.81% ± 0.011. As the average accuracy and standard deviations (the values after ± signs) show the model was highly accurate and stable.

**Figure 3:**
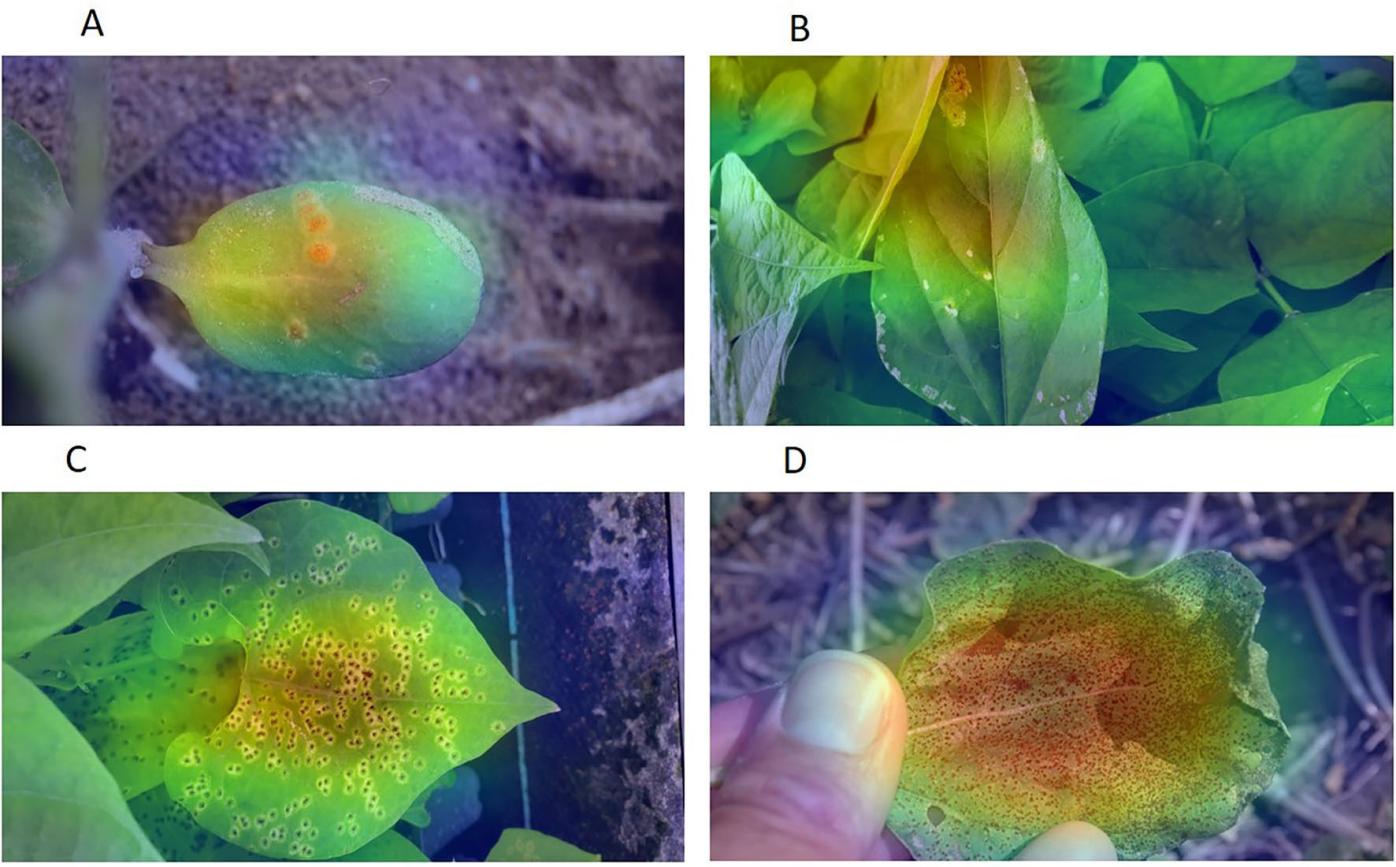
Heat map generated using Gradient-weighted Class Activation Mapping (Grad-CAM) method. The red and blue regions highlight the most and the least discriminative regions, respectively. (**A**) field pea, (**B**) and (**C**) dry bean, and (**D**) sunflower.

Visual demonstrations were generated using Gradient-weighted Class Activation Mapping (Grad-CAM) with EfficientNetB4 as the base model to indicate how deep learning algorithms make decisions in differentiating rust-infested from healthy tissue (Fig. 3). The red area refers to the most important discriminative regions where the model pays the highest attention and the blue areas are the least critical. As Fig. 3A indicates the model mainly focuses on rust postulates and not the background or other injuries such as the one on top right corner of the leaf. Figure 3B shows the precision of the model in focusing on the leaf with rust pustulate while it is surrounded by other healthy leaves. Most importantly, the model is also focused on the rust pustulates located on the leaf tip, not insect injuries at the bottom of the leaf tissue. Figure 3C,D also verify that the rust infested areas on the leaves are correctly recognized as discriminative regions.

As a future plan of this study, the rust disease dataset could be expanded by adding more images of tested crops and additional field crops such as wheat and corn. A larger dataset would allow the validation of the top architectures and, subsequently developing tools and/or mobile applications that assist growers and plant pathologists in a fast and cost-effective plant disease diagnosis. Although diseases such as rust could be detectable by naked eyes, it is not practical to screen the whole field for the presence of the disease and therefore, farmers typically spray the entire field when some symptoms occur. Large scale pesticide applications are costly, labor-intensive, time consuming, and most importantly endanger the environmental health and safety. Application of technology and remote sensing is becoming more common in agriculture with the goal of making precision agriculture accessible to a majority of farmers. The first step toward achieving this goal is the development and validation of accurate machine learning models with a high level of generalization. In this study, our aim was to develop a reliable model for the detection of rust disease on several hosts that could be incorporated into drones and/or handheld devices that facilitate precision spraying.

## Conclusion

Image processing and deep learning algorithms have demonstrated encouraging results in differentiating healthy and infected plants at different stages of the disease progress. In this study, the ability of four different pre-trained CNN models including Xception, ResNet50, EfficientNetB4, and MobileNet were evaluated to detect rust disease on three commercially important field crops. Images from greenhouse and field were used in the training and testing the models to represent the variation of the natural conditions. The performance of the models was evaluated using two important hyperparameters, i.e., learning rate and optimization algorithm. EfficientNetB4 trained by Adam optimizer and learning rate of 0.001 was the most accurate model for discriminating healthy and rust-infested tissues with the average accuracy of 94.29%. These results demonstrated that EfficientNetB4 could be a reliable model to detect rust on several host species and therefore be incorporated into tools and devices used in precision management of the disease such as pesticide spraying drones and robots. Intelligent spray systems are a major component of precision agriculture that results in reducing pesticide applications and protecting environmental and human health.

## Materials and methods

### Image dataset

**Figure 4:**
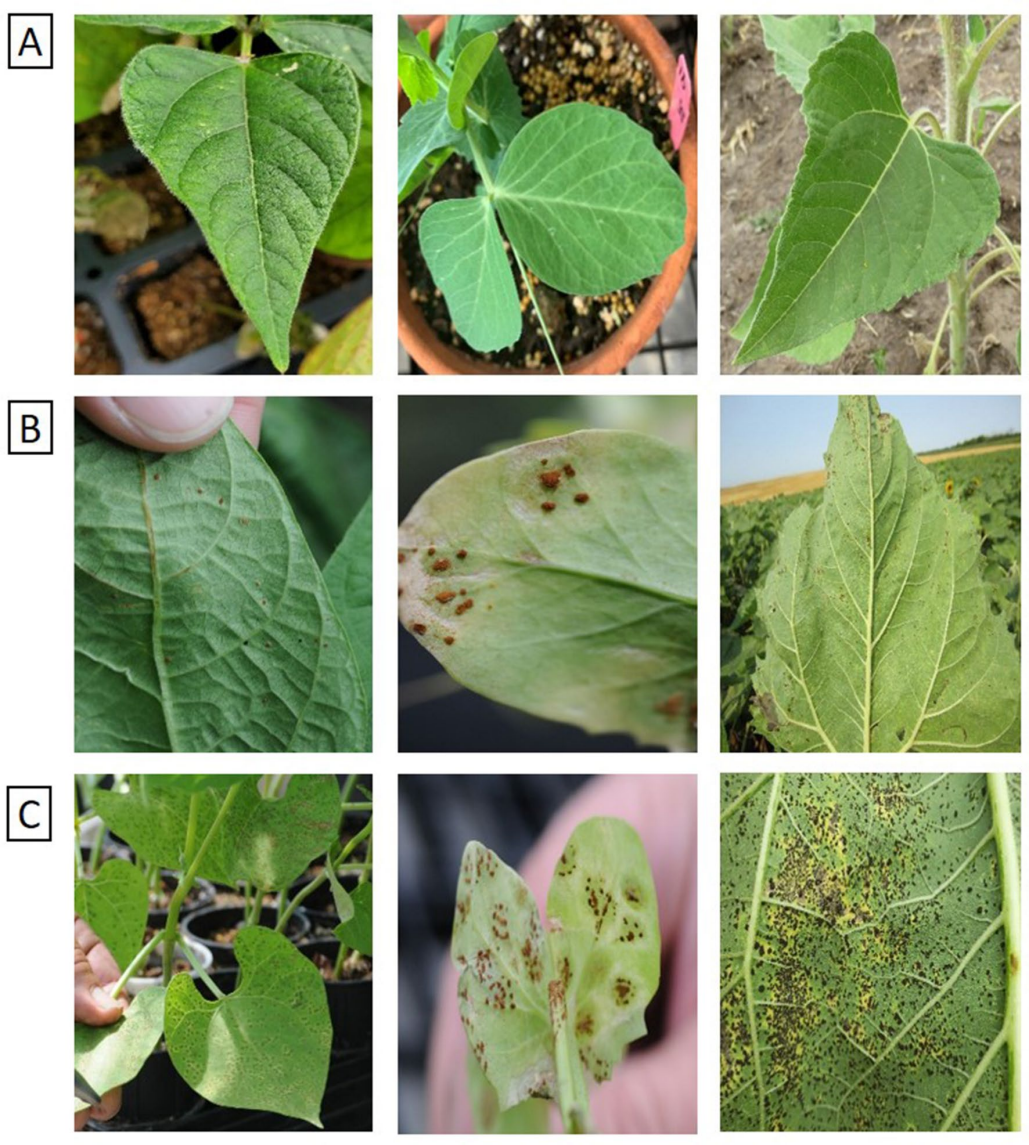
Examples of images used for training the models: (**A**) from left to right: healthy dry bean, field pea, and sunflower leaves, (**B**) from left to right: slightly infected dry bean, field pea, and sunflower leaves, (**C**) from left to right: highly infected dry bean, field pea, and sunflower leaves.

Three crops, including sunflower, dry bean, and field pea, which are susceptible to rust pathogens, were used in this study. Images with different backgrounds and magnitudes were taken preliminary in North Dakota, USA, between 2007 and 2020 under greenhouse and agricultural field environments. The presence of three rust species, including *Puccinia helianthi* on sunflower, *Uromyces appendiculatus* on dry bean, and *U. viciae-fabae* on field pea, on infected plants was visually verified. A total of 1764 images (907 healthy and 857 infected) from three crops were collected and pooled for data analysis where 70% of the photos were used for training. A subsample of healthy and infected plants of three crops is presented in Fig. 4.

All methods were performed in accordance with the relevant guidelines, regulations, and legislation. No animals or human participants were used in this study. Further, plant materials were not collected/planted; only images were taken under field and greenhouse conditions. All study methods followed North Dakota State University guidelines.

### Pre-trained CNN models

In this study, the transfer learning approach using four pre-trained CNN architectures, including Xception, ResNet50, EfficientNetB4, and MobileNet-V2 were adopted for the detection of rust disease on sunflower, field pea, and dry bean. In transfer learning, the model could use previously learned knowledge from other tasks to solve a new problem^55^. Since in transfer learning, the models are already trained on a massive dataset, compared with training a model from scratch, less data is required, and also it could save training time and improve the model performance. In this study, all the models were fine-tuned to diagnose the rust disease using pre-trained weights of the models on the ImageNet dataset which has approximately 1.4 million images in 1000 classes^56^. Further, to adjust the models to our task, which is a binary classification (healthy versus infected), the fully connected layers of the CNN pre-trained models were changed to two, which represents the dimensionality of the output space. The “Sigmoid” function was chosen as the activation function, and “binary cross-entropy” was selected as the loss function.

**Figure 5:**
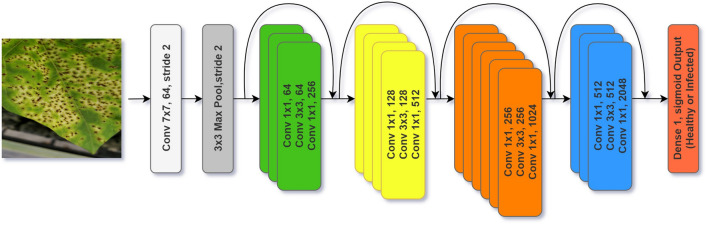
Residual networks (ResNet-50) architecture with a sample input image and 16 residual blocks.

**Figure 6:**
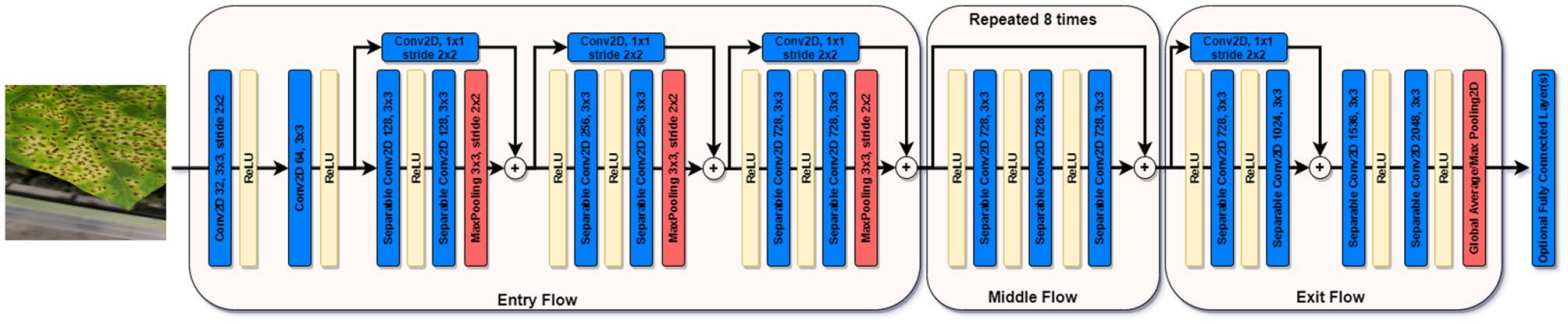
The architecture of the Xception model with a sample input image and 14 modules.

**Figure 7:**
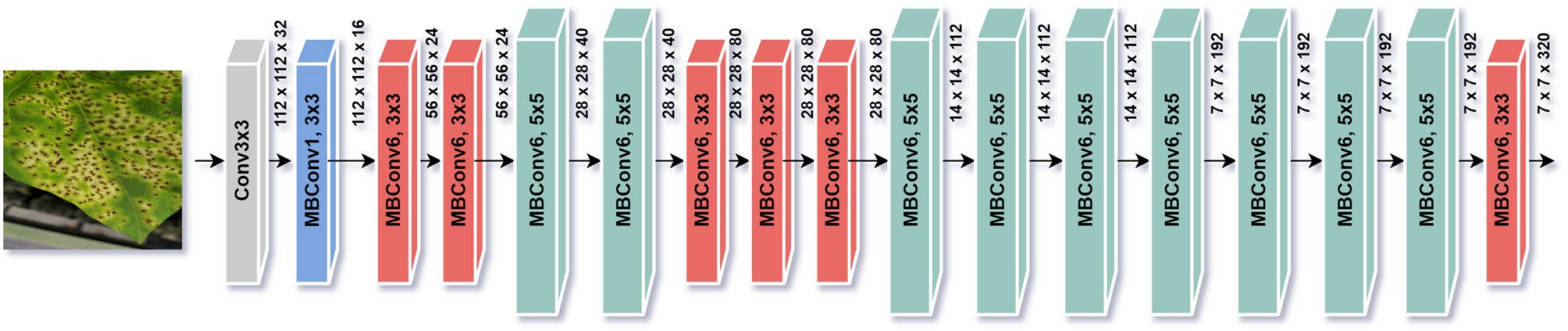
EfficientNet basic architecture with a sample input image and 18 convolution layers.

**Figure 8:**
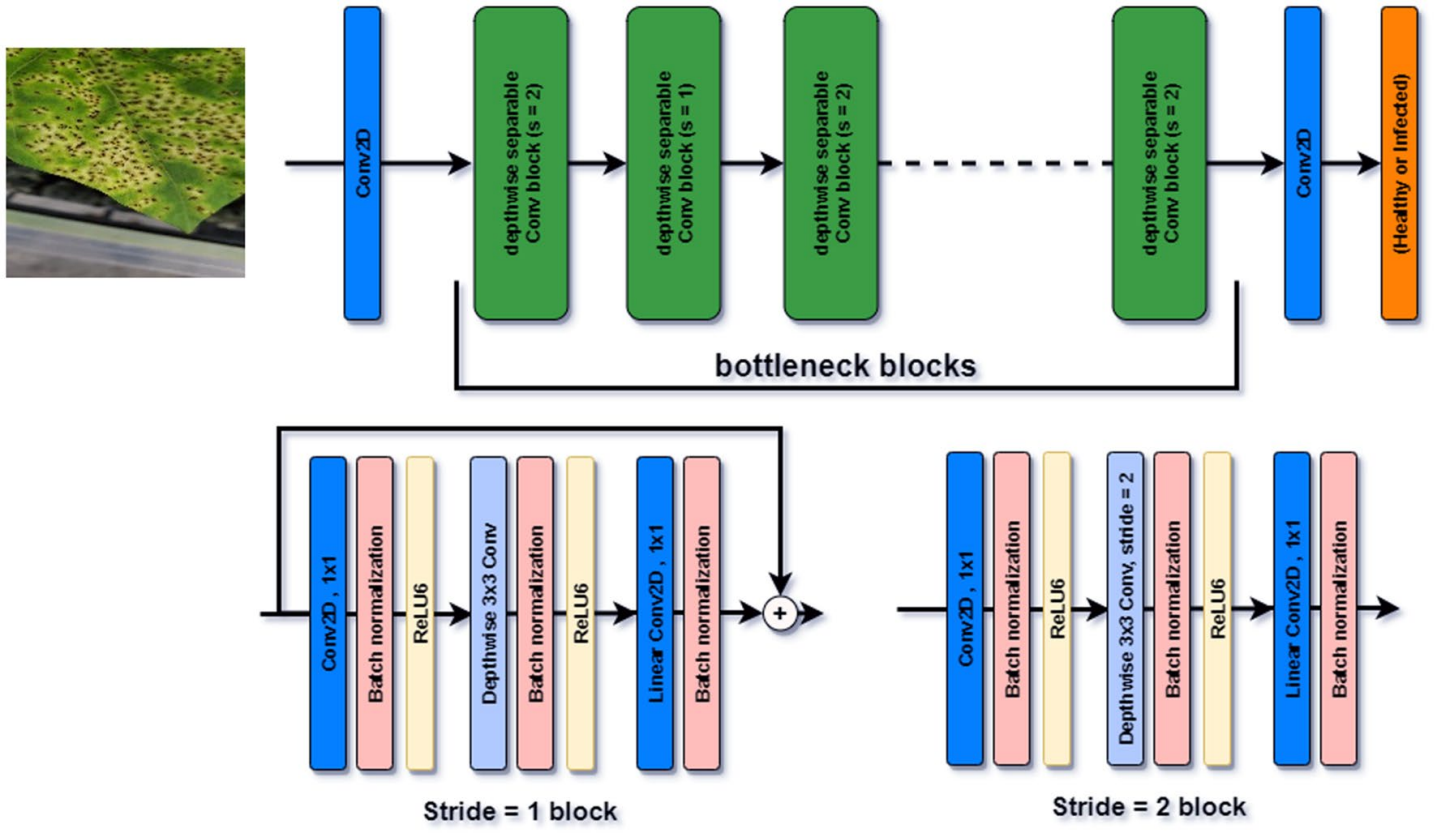
The architecture of the MobileNetV2 with a sample input image and 19 residual bottleneck layers.

The architecture of ResNet50 with a total of 16 residual blocks is represented in Fig. 5. The architecture of the Xception model is shown in Fig. 6. The architecture of EfficientNetB4 with dimensions of 224 × 224 of input images, three channels, and an initial 3 × 3 kernel size convolution is shown in Fig. 7. The architecture of MobileNetV2 includes an initial fully convolution layer with 32 filters, followed by 19 residual bottleneck layers is represented in Fig. 8. The details of the tested pre-trained CNN models’ architecture could be found in the supplementary Note 1.

Further, Grad-CAM^57^ was used to identify the regions of an input picture that have the greatest effect on the classification score. The Grad-CAM method relates the use of the gradients of the classification score to the final convolutional feature map. Locations with a high value of this gradient reflect those with the most data dependence in the final score.

### Model evaluation metrics

The performance of the four deep CNN models was evaluated using some well-known metrics, including accuracy, true negative rate, precision, true positive rate, F-score, and AUC-ROC score. The confusion matrix was used to calculate the performance metrics of the models.

The definitions of these parameters are provided in the next section.

1 $$ {\text{Accuracy}} = { }\frac{{\left( {{\text{TP}} + {\text{TN}}} \right)}}{{\left( {{\text{TP}} + {\text{TN}} + {\text{FP}} + {\text{FN}}} \right)}} $$

2 $$ {\text{True}}\,{\text{ negative}}\,{\text{ rate}} = { }\frac{{{\text{TN}}}}{{{\text{TN}} + {\text{FP}}}} $$

3 $$ {\text{Precision}} = { }\frac{{{\text{TP}}}}{{{\text{TP}} + {\text{FP}}}} $$

4 $$ {\text{True }}\,{\text{positive }}\,{\text{rate}} = { }\frac{{{\text{TP}}}}{{{\text{TP}} + {\text{FN}}}} $$

5 $$ {\text{F}}_{{{\text{score}}}} = 2 \times \frac{{{\text{Precision}} \times {\text{True}}\,{\text{ positive}}\,{\text{ rate}}}}{{{\text{Precision}} + {\text{True}}\,{\text{ positive}}\,{\text{ rate}}}} $$

where TP, TN, FP, and FN represent true positives, true negative, false positives, and false negative, respectively. Based on the Eqs. (1–6), the accuracy of a model is defined as the number of all correct predictions divided by the total number of predictions. True negative rate or specificity, is defined as the correct classification of negative instances, and precision, also known as sensitivity, indicates the total number of correctly classified positive observations. True positive rate or recall refers to the proportion of correctly classified instances, and the F-Score, or F1 score, is the harmonic mean of precision and true positive rate and can be an indicator of the model robustness. In addition, the ROC score represents the performance of the model or diagnostic ability, and it refers to the prediction of the positive instances where the actual observations are positive. It has been reported that the ROC score could be a better comparison measure than F-Score, specifically where class distribution is unbalanced, and the latter might become skewed towards the positive class. Lastly, AUC-ROC indicates the ability of a parameter in the separation of two classes (infected and healthy, in this case).

## Experiment setup

To evaluate the efficiency of deep learning models for detecting rust disease in this study, the experiment was conducted using three different crops, including sunflower, dry bean, field pea and the performance of four well-known pre-trained CNN architectures were compared. All models mentioned above were trained and tested on 70% and 30% of the dataset, respectively. The experiment was implemented on Windows10 using the Keras framework with Tensorflow-GPU v2.6.0 as backend on a GPU-enabled workstation with NVIDIA GeForce GTX 1080 8 GB GDDR5.

Considering that optimizer plays a crucial role in changing the attributes of the models, pre-trained CNN models were trained and tested with four different optimizers including Adam, SGD, RMSprop, and Ftrl. Moreover, since the learning rate is one of the most important hyperparameters^52^ that needs to be tuned to achieve optimum performance, we evaluated the performance of the models using three different learning rates including 0.01, 0.001, and 0.0001. Supplementary Note 2 provides information and related references about the tested hyperparameters.

Other hyperparameters were not benchmarked due to the following reasons: batch size: the computer used in this study had GPU of 1080 which could support a batch size of up to 32; epoch number: the top models were mainly stable across tested epoch numbers. Therefore, higher epoch numbers were not tested (Figs. 1, 2); early stopping: to ensure about the stability of the models across a range of epoch numbers, we did not activate early stopping as it stops the model if there is no improvement in the training at a specific epoch number; Image size: smaller size images were not used due to the small size of the rust pustules; image size: the maximum image size (224 × 224) that our hardware could support was used; depth of model: the depth of the network is fixed for pre-trained models.

## Supplementary Information

Supplementary Information.

## Data Availability

A great part of the data that supports the findings of this study are available from the American Phytopathological Society (APS) but restrictions apply to the availability of these data, which were used under license for the current study (Authors who transfer images to APS retain the ability to use the materials for their individual research, manuscript submission, or Extension activities). Therefore, these data are not publicly available. Data are however available from the corresponding author upon request and with permission of the APS.
