# Supplementary material for: Application of image processing and transfer learning for the detection of rust disease
Source: Sci Rep. 2023 Mar 29;13:5133. doi: 10.1038/s41598-023-31942-9 (PMC10060580; doi:10.1038/s41598-023-31942-9)
Supplement: Supplementary file 1 — Supplementary Information. [file 41598_2023_31942_MOESM1_ESM.docx]

**Supplementary Table S1:** Comparisons between the methodologies and results of the present study and those of previously published studies reporting top classifiers and convolutional neural network models in the classification of binary (healthy vs. infected) and multiple-class (several diseases) plant disease datasets.

| **Study** | **Objective** | **Image acquisition methods**  **(images format/source of data/background and uniformity of images)** | **Tested models** | **Highest accuracy (corresponding model)** |
| --- | --- | --- | --- | --- |
| Present study | Distinguishing rust-infested and healthy tissue using convolutional neural network models | RGB images/ collected by authors in the field and greenhouse/ diverse background and image acquisition conditions | -Xception  -ResNet50  -EfficientNetB4  -MobileNet | 94.3% (EfficientNetB4) |
| Jin et al. ^1^ | Classification of healthy and diseased wheat heads infested with *Fusarium* head blight using convolutional neural network | Hyperspectral images/ wheat head photos collected by authors under field conditions/ uniform black background | -One-dimension convolution neural network (1D-CNN)  -Two-dimensional convolutional neural network (2D-CNN-LSTM, 2D-CNN-GRU, 2D-CNN-BidGRU, 2D-CNN-BidLSTM) | 74% (two-dimensional convolutional neural network= 2D-CNN-BidGRU) |
| Chen et al. ^2^ | Classification of rice diseases using deep neural network | RGB images/ PlantVillage public dataset and data collected by authors in field /close-up uniform background images in PlantVillage, diverse background images in the authors’ dataset | - Inception V3  - VGGNet-19  - DenseNet-121  - NASNetMobile  - MobileNet-V2  - SE-MobileNet | 99.8% in PlantVillage and 99.3% in the authors’ dataset (SE-MobileNet) |
| Chowdhury et al. 2021^3^ | Binary (infected or healthy) and multiple-class (6 disease or 10 diseases) detection of tomato leaf diseases using convolutional neural network | RGB images/ PlantVillage public dataset/ close-up uniform background images | -EfficientNet-B0  -EfficientNet-B4  -EfficientNet-B7 | 99.6% for binary (EfficientNet-B7)- 99.1% for six-class (EfficientNet-B7), and 99.9% for ten-class data (EfficientNet-B4) |
| **Study** | **Objective** | **Image acquisition method**  **(images format/source of data/background and uniformity of images)** | **Tested models** | **Highest accuracy (corresponding model)** |
| Zhang et al. 2018 ^4^ | Identification of tomato leaf disease (infected or healthy) using deep convolutional neural network | RGB images/ PlantVillage public dataset/ close-up uniform background images | -AlexNet  -GoogLeNet  -ResNet | 97.3% (ResNet) |
| Pardede et al. 2020 ^5^ | Identification of multiple-class plant diseases using convolutional neural network models | RGB images/ PlantVillage public dataset and low-resolution image data collected by authors /close-up uniform background images in both datasets | -VGGNet  -AlexNet  -Resnet  -Xception  -MobileNet  -ComNet | 96.6% in PlantVillage and 86.2% in authors’ dataset (ComNet) |
| Sagar and Dheeba 2020 ^6^ | Identification of multiple-class plant diseases using deep neural networks | RGB images/ PlantVillage public dataset/ close-up uniform background images | -VGG16  -ResNet50  -InceptionV3 -InceptionResNet -DenseNet169 | 98% (ResNet50) |
| Xie et al. 2017 ^7^ | Early detection of gray mold disease on tomato leaves (differentiating infected and healthy tissue) using classifiers | Hyperspectral image/ tomato leaf images collected by authors greenhouse conditions/ black background | - K-nearest neighbor (KNN)  -C5.0  - Features ranking K-nearest neighbor (FR-KNN) | 97.2% (FR-KNN) |
| Zhang et al. 2019 ^8^ | Detection of wheat rust diseases in the field using deep convolutional neural network | High spatial resolution hyperspectral images captured with UAVs/ Authors’ dataset/ Arial photos in a similar frame and minimum margins | -Multiple Inception-Resnet  -Random forest | 85% (multiple Inception-Resnet) |
| **Study** | **Objective** | **Image acquisition method**  **(images format/source of data/background and uniformity of images)** | **Tested models** | **Highest accuracy (corresponding model)** |
| Wang et al. 2017 ^9^ | Diagnosing the severity of apple black rot images using deep convolutional neural networks | RGB images/ PlantVillage public dataset/ close-up uniform background images | - VGGNet  - Inception-v3  - ResNet50 | 90.4% (VGG16) |
| Atila et al. 2021 ^10^ | Classification of plant leaf diseases using EfficientNet deep learning architecture | RGB images/ PlantVillage public dataset/ close-up uniform background images | -AlexNet  -ResNet50  -VGG16  - Inception V3  - EfficientNet (B0 to B7) | 99.97% (EfficientNet-B4) |
| Mi et al. 2020 ^11^ | Grading wheat stripe rust disease using a novel deep learning network | RGB images/ WSRgrading dataset collected in the natural field condition/ diverse background; backgrounds were mostly cropped | - DenseNet  - C-DenseNet  - ResNet | 97.99% (C-DenseNet) |
| Fuentes et al. 2017 ^12^ | Detect diseases and pests in tomato plants using deep-learning-based approach | RGB images/ collected by authors in the field and greenhouse/ diverse background and image acquisition conditions | Several models in the following families:  - Faster R-CNN  - R-FCN  - SSD | 80-91% depending on the feature extraction method (VCG-16 in Faster R-CNN family and ResNet-50 in R-FCN family) |

**References:**

Jin, X., Jie, L., Wang, S., Qi, H.J. & Li, S.W. Classifying wheat hyperspectral pixels of healthy heads and Fusarium head blight disease using a deep neural network in the wild field. *Remote Sensing*, **10(3)**, 395 (2018).

1. Chen, J., Zhang, D., Suzauddola, M., Nanehkaran, Y. A. & Sun, Y. Identification of plant disease images via a squeeze‐and‐excitation MobileNet model and twice transfer learning. *IET Image Process.* **15,** 1115-1127 (2021).
2. Chowdhury, M. E. *et al.* Automatic and Reliable Leaf Disease Detection Using Deep Learning Techniques. *AgriEngineering* **3**, 294-312 (2021).

Zhang, S., Zhou, H. & Zhang, L. Recent Machine Learning Progress in Image Analysis and Understanding. *Advances in Multimedia*, 1-2 (2018).

1. Pardede, H. F. *et al.* Plant diseases detection with low resolution data using nested skip connections. *J. Big Data.* **7**, 1-21 (2020).
2. Sagar, A. & Dheeba, J. On using transfer learning for plant disease detection. *bioRxiv* (2020).

Xie, C., Yang, C. & He, Y. Hyperspectral imaging for classification of healthy and gray mold diseased tomato leaves with different infection severities. *Computers and electronics in agriculture*, **135**, 154-162 (2017).

Zhang, X. et al. A deep learning-based approach for automated yellow rust disease detection from high-resolution hyperspectral UAV images. *Remote Sensing*, **11(13)**, 1554 (2019).

Wang, G., Sun, Y. & Wang, J. Automatic image-based plant disease severity estimation using deep learning. *Comput. Intell. Neurosci.* **2017** (2017).

Atila, Ü., Uçar, M., Akyol, K. & Uçar, E. Plant leaf disease classification using EfficientNet deep learning model. *Ecol. Inform.* **61**, 101182 (2021).

1. Mi, Z., Zhang, X., Su, J., Han, D. & Su, B. Wheat stripe rust grading by deep learning with attention mechanism and images from mobile devices. *Front. Plant Sci.* **11** (2020).

Fuentes, A., Yoon, S., Kim, S. C. & Park, D. S. A robust deep-learning-based detector for real-time tomato plant diseases and pests recognition. *Sensors* **17**, 2022 (2017).

**Supplementary Note 1:**

In this study, the transfer learning approach using four pre-trained CNN architectures, including Xception, ResNet50, EfficientNetB4, and MobileNet-V2 was used. The following describes the architecture of each model and represents the related references:

***ResNet****:* Each layer of CNN learns low or high-level features throughout the training process. Deeper neural networks, however, have difficulties like vanishing gradients and problems with degradation. The ResNet^1^ employs shortcut connections parallel to each stack of convolution layers which are called identity connections, to minimize the complexity of training and degradation. This model attempts to learn residues instead of learning features. The introduction of the identity connections to the network adds no extra parameters to the final network. As a result, the computing complexity for ResNet and the basic deep network would be the same, which results in considerable performance improvement in training and generalization error^1^. The feedforward building block with the identity connection in ResNet architecture is shown in the following figure (Supplementary Note 1, Fig. 1).


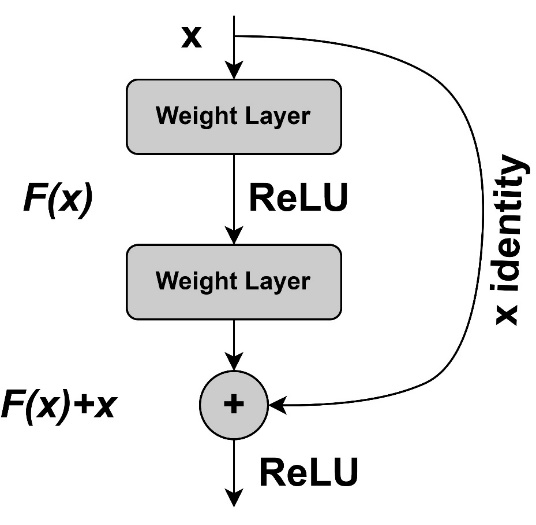


**Supplementary Note 1, Figure 1:** **Feedforward building block with the shortcut connection in residual networks (ResNet) architecture**


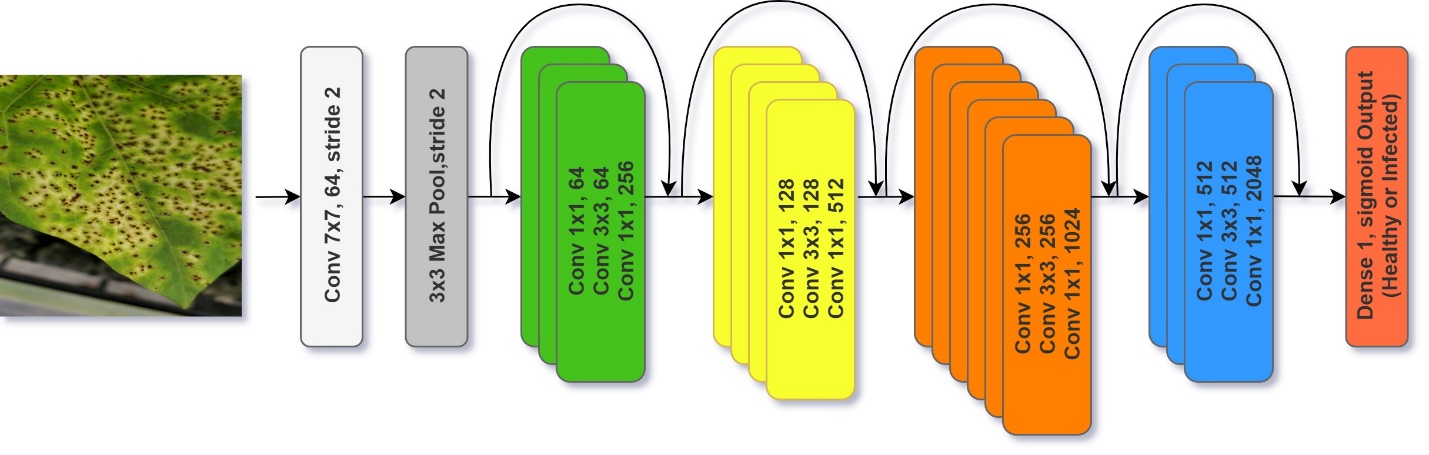
 In this study, ResNet50 with a depth of 50 layers was evaluated to classify the infected and healthy plants. The architecture of ResNet50 with a total of 16 residual blocks is represented in the following figure (Supplementary Note 1, Fig. 2).

**Supplementary Note 1, Figure 2:** **Residual networks (ResNet-50) architecture with a sample input image and 16 residual blocks**

The network accepts an input image with dimensions of 224×224 with three channels. ResNet architecture operates an initial 7×7 kernel size convolution and 3×3 kernel size max-pooling. Following that, the first stage of the network begins with three residual blocks containing three layers each. The kernels in three layers of the block are 64, 64, and 128, respectively, which the identity connections are denoted by the curved arrows in Supplementary Note 1, Figure 2. As we proceed through the stages, the channel width and the input size are doubled and reduced by half, respectively. With an input image size of 224×224, the total trainable parameters of ResNet50 architecture are over 23 million^1^.

***Xception****:* The Xception^2^ is a CNN model that is an improved version of the Inception model proposed by Google researchers^3^. In the Xception model, the standard Inception architecture is replaced with depthwise separable convolutions (DSC). The basic concept behind the DSC is replacing conventional convolutions, which tries to learn features across a joint "space-cross-channels domain with two simplified steps: a spatial feature learning step and a channel combination step^4^. Hence, compared to classical convolutions, which is a point-by-point convolution, DSC will produce fewer parameters which not only decrease the chance of overfitting but are expected to have a substantially shorter computation time. Moreover, similar to ResNet architecture, in the Xception model, a residual connection is added within each module. The residual connections can lead to several advantages, such as increasing the depth of the architecture by generating fewer parameters, increasing the speed of the training, reducing the impact of vanishing gradient on the network, and improving the accuracy of the model. The Xception architecture is composed of 36 convolutional layers in three sections of entry flow, middle flow, and exit flow. The input images initially pass via the input flow, then through the middle flow (repeated eight times), and lastly through the exit flow. Except for the first and last modules, the 36 convolutional layers are divided into 14 modules, all of which contain linear residual connections surrounding them^2^.

***EfficientNetB4****:* CNNs are frequently scaled in depth^1^ and width^5^ for improving performance and accuracy. For example, wide residual networks (WRNs)^5^ scale up the network from WRN-40-4 to WRN-28-^1^0 by shallowing the depth but widening the convolutional layers. The first and second numbers in the WRNs are the depth and the widening factor of the model. Compared to ResNet and WRNs, EfficientNet^6^ delivers more efficient results through the scaling of the model by uniformly scaling its depth, width, and resolution. The EfficientNetB0 with 18 convolution layers is the most basic configuration where the main building block of its architecture is the mobile inverted bottleneck (MBConv)^7^. In each MBConv block, channels are squeezed in resolution to reduce the feature map size but increased in width to improve accuracy, and then the bottlenecks are connected directly to each other with far fewer channels than the expansion layer^6^. EfficientNet benefits from the Swish activation function, instead of rectified linear unit (ReLU) activation. Swish is a smooth curve (following equation and Supplementary Note 1, Fig. 3) that its output is generally smoother than ReLU. This offers advantages regarding converging toward minimum loss when optimizing the model.

$$f\left( x \right)=x*sigmoid\left( x \right)=x*\left( 1+e^{-x} \right)^{-1}$$

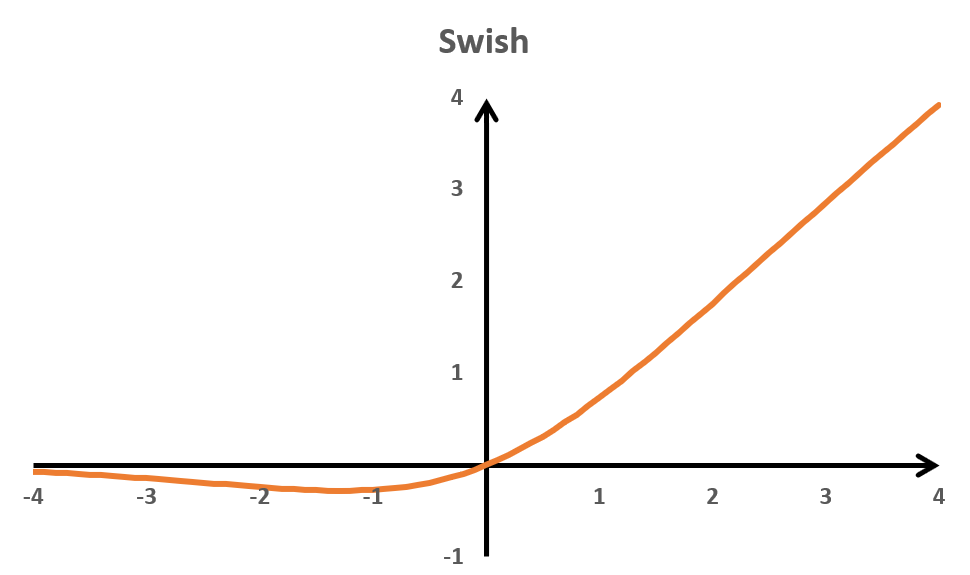


**Supplementary Note 1, Figure 3:** **Swish activation function graph**

The first stage of scaling up the EfficientNetB0 is a grid search to define the connection between the dimensions of the baseline network under a given resource restriction. This stage determines the appropriate scaling factors for depth, width, and resolution. In the next stage, the coefficients are applied to EfficientNetB0 to establish EfficientNet-B1 to B7. This study evaluated EfficientNetB4 with 19 million parameters, almost the size of Xception and ResNet50, to build a fair comparison between these models^6^. The network accepts an input image with dimensions of 224 × 224 with three channels and operates an initial 3×3 kernel size convolution.

***MobileNetV2***: this architecture was introduced by Google and is an efficient model for mobile and embedded vision applications with limited computational resources^7^. MobileNet is built on a simplified architecture to build a lightweight deep neural network DSC^8^. As explained above, DSC is a kind of factorized convolutions in which a standard convolution is factorized into a Depthwise Convolution and a 1×1 pointwise convolution^8^. MobileNetV2^7^ is an improved version of MobileNetV1 with some minor modifications. It also uses DSC as its core building block with two new features, including linear bottlenecks between the layers and shortcut connections between the bottlenecks. MobileNetV2 can be used for several visual recognition tasks, including classification, object detection, and semantic segmentation. The architecture of MobileNetV2 includes an initial fully convolution layer with 32 filters, followed by 19 residual bottleneck layers. In addition, to maintain the non-linearity, ReLU6 is used due to its robustness when employed with low-precision computations^7^. Further, during the training process, the model employs the kernel size of 3×3 like the other modern networks as well as batch normalization and dropout. One of the main advantages of the MobileNetV2 compared to the older version is the fewer number of parameters generated by the model during the training process^7^.

**References:**

1 He, K., Zhang, X., Ren, S. & Sun, J. Deep residual learning for image recognition. in *Proceedings of the IEEE conference on computer vision and pattern recognition (CVPR).* 770-778 (2016).

2 Chollet, F. Xception: Deep learning with depthwise separable convolutions. in *Proceedings of the IEEE conference on computer vision and pattern recognition (CVPR).* 1251-1258 (2017).

3 Szegedy, C., Vanhoucke, V., Ioffe, S., Shlens, J. & Wojna, Z. Rethinking the inception architecture for computer version. in *Proceedings of the IEEE conference on computer vision and pattern recognition (CVPR).* 2818-2826 (2016).

4 Kaiser, L., Gomez, A. N. & Chollet, F. Depthwise separable convolutions for neural machine translation. *arXiv preprint arXiv:1706.03059* (2017).

5 Zagoruyko, S. & Komodakis, N. Wide residual networks. *arXiv preprint arXiv:1605.07146* (2016).

6 Tan, M. & Le, Q. Efficietnet: Rethinking model scalilng for convolutional neural networks. in *International Conference on Machine Learning* 6105-6114 (PMLR) (2019).

7 Sandler, M., Howard, A., Zhu, M., Zhmoginov, A. & Chen, L.-C. Mobilenetv2: Inverted residuals and linear bottlenecks. in *Proceedings of the IEEE conference on computer vision and pattern recognition (CVPR).* 4510-4520 (2018).

8 Howard, A. G. *et al.* Mobilenets: Efficient convolutional neural networks for mobile vision applications. *arXiv preprint arXiv:1704.04861* (2017).

**Supplementary Note 2:**

Optimizer and learning rate are important factors in developing and validating pre-trained convolutional neural network (CNN) models; therefore, we tested three different learning rates including 0.01, 0.001, and 0.0001, and four different optimizers including Adam, SGD, RMSprop, and Ftrl. The following represents descriptions of tested optimizers’ algorithms:

**Stochastic Gradient Descent (SGD)**: SGD is a simple and efficient algorithm that can handle massive datasets by updating the model parameters using only a small subset of the data at each iteration. This allows for faster convergence and lower computational costs compared to traditional gradient descent methods. In SGD parameters are updated at each training sample. It is recommended to randomly select training samples in advance of training in each epoch. For large training datasets, this method is both more memory-efficient and faster than Batch Gradient Descent (BGD). However, because SGD is updated frequently, it takes noisy steps in the direction of the answer, which makes the convergence behavior highly unstable^1^. The formula for Stochastic Gradient Descent (SGD) is:

θ = θ - α * ∂L/∂θ

where θ represents the current model parameters, α is the learning rate, and ∂L/∂θ represents the gradient of the loss function L with respect to the model parameters θ, evaluated at a single training example. This update is repeated for each training example in the dataset^2^.

**Root Mean Square Propagation (RMSProp)**: RMSProp is an adaptive stochastic algorithm that is widely used for training deep neural networks. It is a modified version of Adagrad and works by accumulating the gradient through an exponentially weighted average. However, unlike Adagrad, RMSProp discards past gradient knowledge and preserves only the current gradient knowledge. This modification helps prevent the learning rate from becoming too small during training, which can lead to slow convergence or getting stuck in local minima. Overall, RMSProp is a popular and effective optimization technique for training deep neural networks. Provide the following formula for RMSProp:

E[g^2^]_t_ = 0.9E[g^2^]_t-1_ + 0.1g^2^_t_

θ_t+1_ = θ_t_ - (η / √(E[g^2^]_t_ + ε)) g_t_

where: E[g^2^]t is the exponentially weighted average of the squared gradients, g^2^_t_ is the squared gradient at time t, θ_t_ is the parameter vector at time t, η is the learning rate, γ is the decay rate, which is suggested to be set to 0.9 by Hinton, g_t_ is the gradient at time t, ε is a small constant (e.g. 10^-8^) to avoid division by zero^3^.

**Adaptive Moment Estimation (Adam)**: Adam is a commonly used optimization technique in deep learning. It is a representation of the latest trends in optimization and utilizes the Hessian matrix, which uses a second-order derivative. Adam is specifically designed for training deep neural networks, and it has the advantages of being more memory-efficient and requiring less computational power. Adam calculates an adaptive learning rate for each parameter in the model. It combines the benefits of both Momentum and RMSprop by using squared gradients to scale the learning rate, similar to RMSprop, and by using the moving average of the gradient, similar to Momentum^4^. The equation for this optimizer is:

w_ijt_ = w_ijt-1_ - η * E[δ^2^]t^(-1/2)^ + ε * E[δ^2^]t^(-1/2)^

where w_ijt_ is the weight for the i-th layer and j-th node at time t, _wijt-1_ is the weight at the previous time step, η is the learning rate, E[δ^2^]t is the moving average of the squared gradient, ε is a small value used to avoid division by zero, and t is the time step^5^.

**Follow-The-Regularized-Leader (FTRL)**: FTRL is particularly useful for large-scale machine learning problems. The FTRL optimizer algorithm computes an update to the model's parameters based on the gradient of the loss function and a regularization term. The regularization term is chosen to encourage sparsity in the solution and to prevent overfitting. McMahan et al. 2013 describe this algorithm in detail^6^.

**References:**

Bottou L. Large-scale machine learning with stochastic gradient descent. in *Proceedings of COMPSTAT’2010*. *Springer,* 177–86 (2010).

Rumelhart, D.E., Hinton, G.E. & Williams, R.J. Learning representations by back-propagating errors. *nature*, ***323*(6088)**, 533-536 (1986).

Ruder, S. An overview of gradient descent optimization algorithms. *arXiv preprint arXiv:*1609.04747 (2016).

Zhang Z. Improved Adam optimizer for deep neural networks. In: *2018 IEEE/ACM 26th international symposium on quality of service (IWQoS). IEEE*, 1–2 (2018).

Alzubaidi, L. et al. Review of deep learning: Concepts, CNN architectures, challenges, applications, future directions. *Journal of big Data*, ***8***, 1-74 (2021).

McMahan, H. B. *et al.* Ad click prediction: a view from the trenches. in *Proceedings of the 19th ACM SIGKDD international conference on Knowledge discovery and data mining* 1222-1230 (2013).
